# Reconstruction of the transmission dynamics of the first COVID-19 epidemic wave in Thailand

**DOI:** 10.1038/s41598-022-06008-x

**Published:** 2022-02-07

**Authors:** Chaiwat Wilasang, Natcha C. Jitsuk, Chayanin Sararat, Charin Modchang

**Affiliations:** 1 Biophysics Group, Department of Physics, Faculty of Science, Mahidol University, Bangkok, 10400 Thailand; 2 Centre of Excellence in Mathematics, CHE, Bangkok, 10400 Thailand; 3 Thailand Center of Excellence in Physics, CHE, 328 Si Ayutthaya Road, Bangkok, 10400 Thailand

**Keywords:** Viral infection, Computational science, Epidemiology

## Abstract

Thailand was the first country reporting the first Coronavirus disease 2019 (COVID-19) infected individual outside mainland China. Here we delineated the course of the COVID-19 outbreak together with the timeline of the control measures and public health policies employed by the Thai government during the first wave of the COVID-19 outbreak in Thailand. Based on the comprehensive epidemiological data, we reconstructed the dynamics of COVID-19 transmission in Thailand using a stochastic modeling approach. Our stochastic model incorporated the effects of individual heterogeneity in infectiousness on disease transmission, which allows us to capture relevant features of superspreading events. We found that our model could accurately capture the transmission dynamics of the first COVID-19 epidemic wave in Thailand. The model predicted that at the end of the first wave, the number of cumulative confirmed cases was 3091 (95%CI: 2782–3400). We also estimated the time-varying reproduction number (*R*_*t*_) during the first epidemic wave. We found that after implementing the nationwide interventions, the *R*_*t*_ in Thailand decreased from the peak value of 5.67 to a value below one in less than one month, indicating that the control measures employed by the Thai government during the first COVID-19 epidemic wave were effective. Finally, the effects of transmission heterogeneity and control measures on the likelihood of outbreak extinction were also investigated.

## Introduction

Coronavirus disease 2019 (COVID-19), caused by severe acute respiratory syndrome coronavirus 2 (SARS-CoV-2), was first identified in Wuhan, China, in late December 2019^1^. The disease then spread rapidly across multiple countries in early 2020. Most of the early imported cases in these countries reported having a history of travel to Wuhan^2^. The severity of the outbreak in these countries varied significantly, depending on the timeliness and effectiveness of state control measures^3^.

Thailand was the first country that reported the first COVID-19 infected individual outside of mainland China^4,5^. The infected patient was reported on 13 January 2020, and the local transmission in Thailand subsequently emerged at the end of January 2020^4^. The number of COVID-19 confirmed cases in Thailand rose rapidly after two large outbreaks at Lumphini Boxing Stadium and at Thong Lo entertainment venue in early March 2020^6,7^. The Thai government then enforced several control measures to prevent and mitigate outbreaks^7–10^. Consequently, the number of locally transmitted COVID-19 cases in Thailand had declined and reached zero on 13 May 2020 and had since remained zero for at least 220 consecutive days^8,10,11^.

In this study, we described the course of the COVID-19 outbreak together with the timeline of the control measures and public health policies employed by the Thai Government during the first wave of the COVID-19 outbreak in Thailand. We also investigated the impact of non-pharmaceutical intervention strategies on the epidemiology of the first wave of COVID-19 in Thailand. Based on the comprehensive epidemiological data, we delineated the full dynamics of the first wave of COVID-19 in Thailand using a stochastic event-based epidemic model and a time-varying reproduction number. In addition, as pointed out by several studies^12–19^, our stochastic model also incorporates individual heterogeneity in infectiousness on SARS-CoV-2 transmission. This allows us to accurately capture the relevant features of superspreading events (SSEs), i.e., a transmission characteristic in which only a few infectious individuals transmit the disease to large numbers of individuals, while most infected individuals infect a few or none^20,21^. Finally, the effects of transmission heterogeneity and control measures on the likelihood of outbreak extinction were also investigated.

## Results

### The first wave of COVID-19 in Thailand

**Figure 1:**
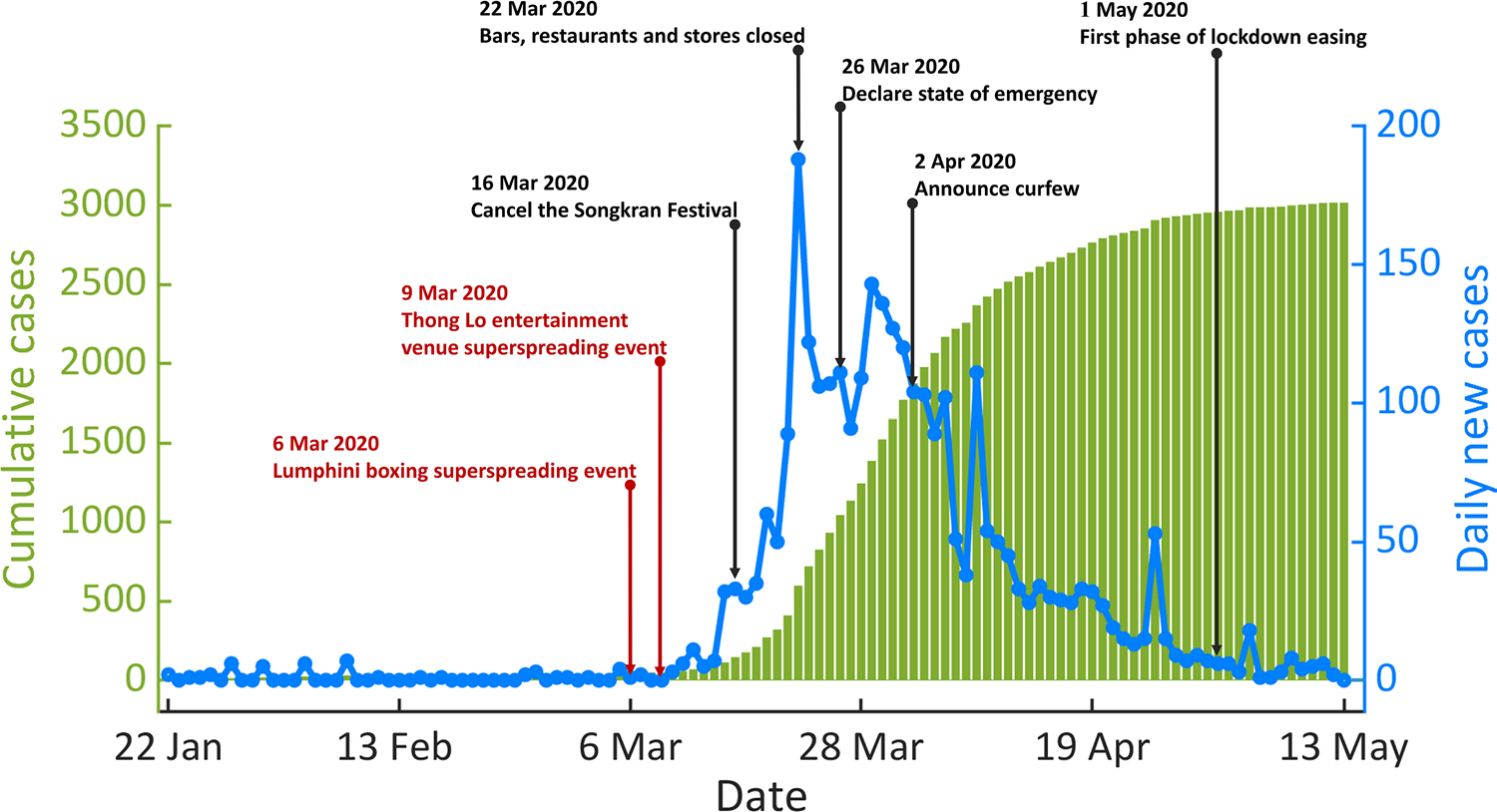
The first wave of COVID-19 outbreak in Thailand. The blue line illustrates the number of daily new locally transmitted cases during the first epidemic wave (from 22 January to 13 May 2020). The green bar shows the corresponding number of cumulative cases. The black arrows indicate the time points at which control measures were implemented, while the time points of the large cluster outbreaks are shown using the red arrows.

On 13 January 2020, Thailand reported the first confirmed case of COVID-19 outside China^4,5,22^. The first detected case was a traveler from Wuhan, China, who had symptom onset on 3 January 2020. Not long after that, the first locally transmitted COVID-19 case in Thailand was reported on 22 January 2020. The Thai government then enforced several control measures to prevent and mitigate outbreaks^7–10^. Figure 1 shows the numbers of daily and cumulative confirmed locally transmitted COVID-19 cases along with some key events in Thailand during the first epidemic wave. At the end of the first epidemic wave (13 May 2020), 3,017 cumulative cases and 56 deaths (1.9%) had been recorded. Among 3017 infected individuals, 2492 (82.6%) are adults (aged 20–59 years), and 332 (11.0%) are elderly (aged over 60 years old)^8,23^.

In Thailand, the COVID-19 transmission during the first epidemic wave was mostly related to large cluster outbreaks in crowded areas^6,7,9^. As shown in Fig. 1, the number of daily new cases increased rapidly after two large cluster outbreaks at the Lumphini boxing stadium (6 March 2020) and at a nightclub in Thong Lo (9 March 2020)^6^. The Thai government then employed several control measures to mitigate the spread of COVID-19. Physical distancing and work-from-home measures were implemented. Nightclubs, restaurants, stores, and schools were ordered to close across the country. Also, the Songkran Festival, i.e., the Thai New Year festival, was canceled to avoid mass gatherings^24,25^. In addition, the government implemented a nationwide curfew (between 10 p.m. to 4 a.m) on 2 April 2020, and all international flights to Thailand were banned starting from 6 April 2020^10,23^. The daily confirmed cases were subsequently decreased, leading to the lockdown easing on 1 May 2020^8,10^. The number of locally transmitted COVID-19 cases during the first epidemic wave in Thailand had reached zero for the first time on 13 May 2020 and had since remained zero for at least 200 consecutive days^8,10,11^.

**Figure 2:**
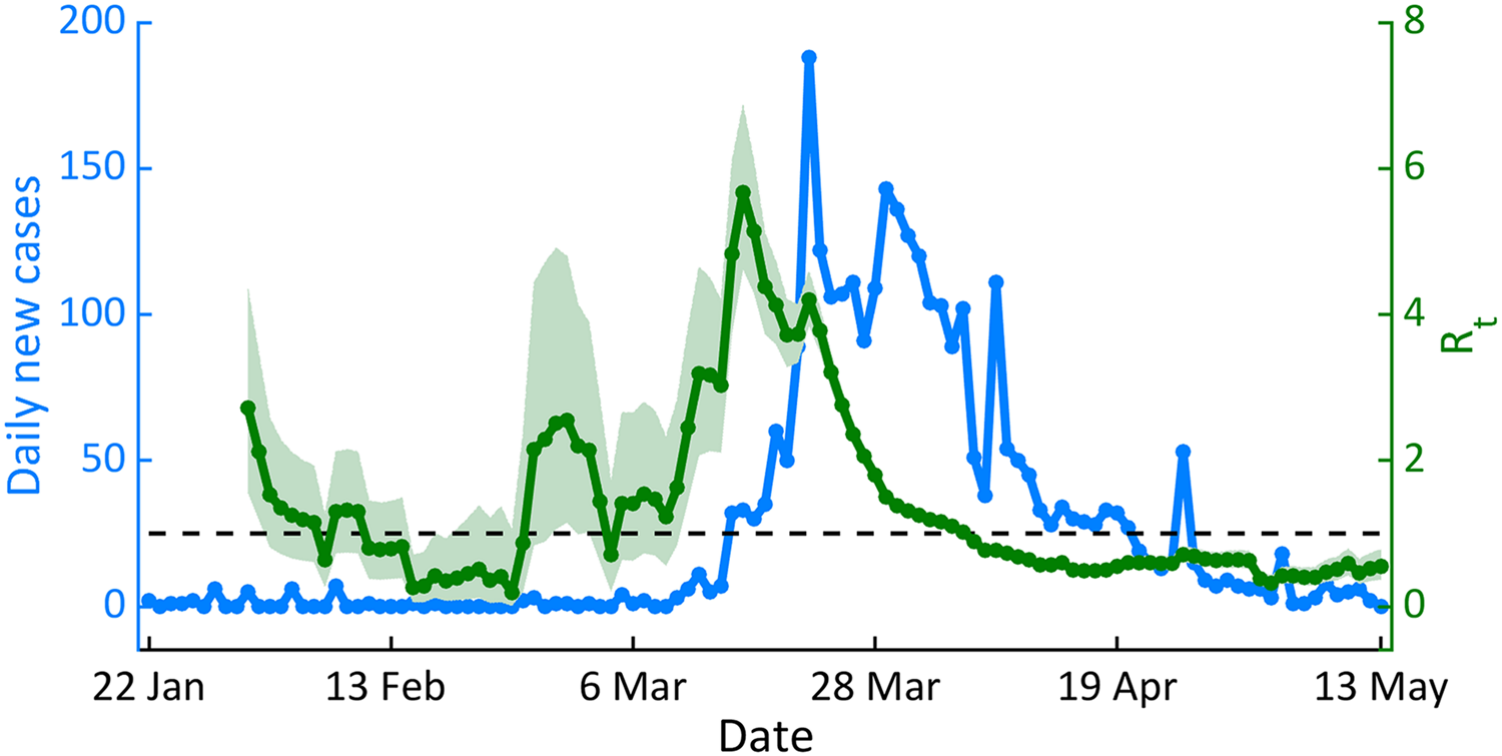
The time-varying reproduction numbers during the first epidemic wave. The solid green line shows the median of $R_{t}$ and the green shaded area indicates the 95% confidence interval (CI). The horizontal dashed black line indicates the $R_{t}$ threshold value of 1. The solid blue line indicates the number of daily new COVID-19 cases.

To evaluate the effectiveness of the implemented control measures, we then estimated the time-varying reproduction number, $R_{t}$, which represents the average number of secondary cases caused by a primary infected individual. A value of $R_{t}$ greater than the threshold value of 1 indicates that the epidemic size is growing at time *t*, whereas $R_{t}$ < 1 indicates that the epidemic size is shrinking at time *t*. The goal of the control measures is, therefore, to reduce $R_{t}$ < 1. As shown in Fig. 2, the $R_{t}$ in the early phase of transmission was around 2.72 (95% CI 1.56–4.35). However, after the large outbreaks at the Lumphini boxing stadium and the nightclub in Thong Lo, the $R_{t}$ increased rapidly and peaked at 5.67 (95% CI 4.62–6.87) on 18 March 2020, which was just before nightclubs, restaurants, and stores were ordered to close. After Thailand had implemented several control measures, the $R_{t}$ decreased to a value < 1 for the first time on 6 April 2020 and then had since remained below one until the end of the first epidemic wave.

### Reconstruction of the first COVID-19 epidemic wave in Thailand

**Figure 3:**
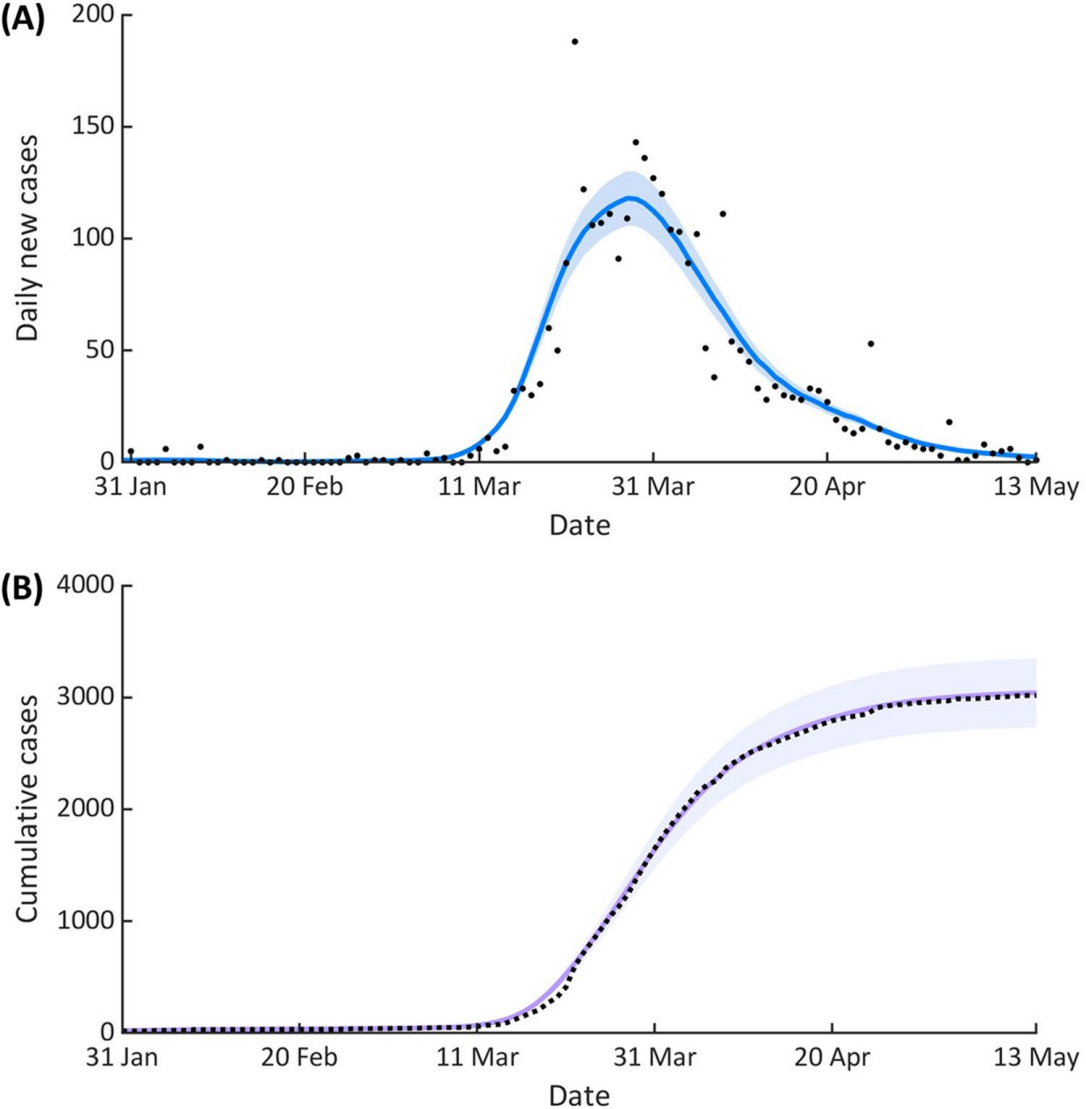
Reconstruction of the first COVID-19 epidemic wave in Thailand. (**A**) The black dots represent the reported daily new cases, while the blue line indicates the average number of daily new cases generated from the model. (**B**) The black dash line represents the number of cumulative cases and the solid line shows the number of cumulative cases obtained from the model simulations. For both (**A**) and (**B**), the shaded areas indicate the 95% CIs. The dispersion parameter $k$ was fixed at 0.14, $q_{S}$ = 0.6 and $q_{A}$ = 0.6.

To reconstruct the transmission dynamics of COVID-19 in Thailand, we used the estimated $R_{t}$ to calculate the time-dependent transmission rate of SARS-CoV-2 in Thailand (see Material and Methods). The comparison between the modeling results and the reported data is shown in Fig. 3. We found that the trends of both daily new cases and cumulative cases generated from the model agree well with the corresponding observed data (R-square = 0.9996). The mean time delay from onset of infectiousness to isolation, representing the effective infectious period of isolated individuals, was estimated at 2.26 days. The model predicted that at the end of the first wave, the number of cumulative confirmed cases was 3,091 (95%CI 2782–3400), which is close to the number of reported cases.

### Characterizing the SARS-CoV-2 transmission dynamics

**Figure 4:**
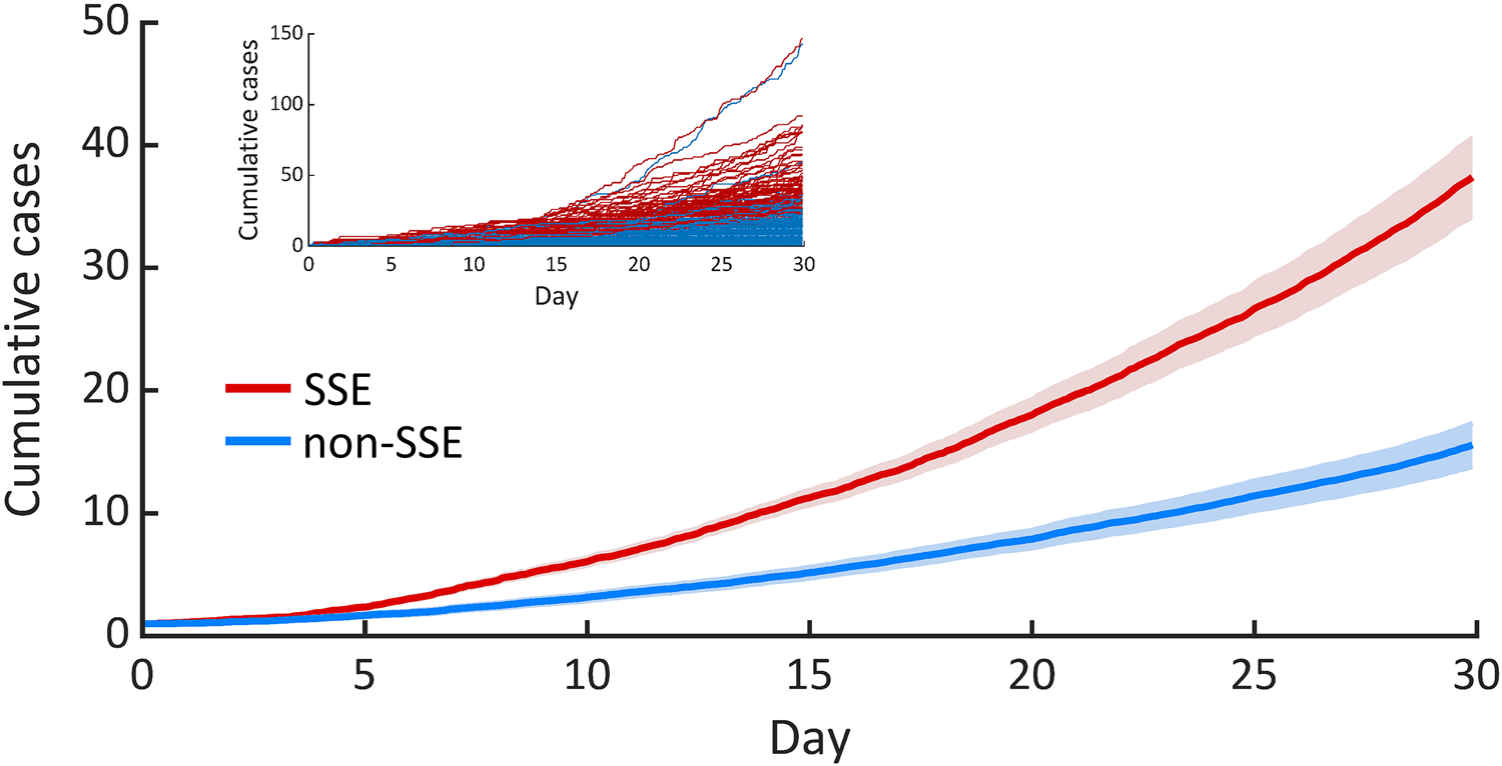
Characteristic of SARS-CoV-2 transmission. The blue and red lines show the average numbers of cumulative cases of non-SSE- and SSE-related simulations, respectively. Shaded areas indicate the corresponding standard error of the mean (SEM). The inset illustrates the individual trajectories of non-SSE- (blue) and SSE- (red) related SARS-CoV-2 transmission.

To characterize the SARS-CoV-2 transmission dynamics, we employed the constructed model to simulate the SARS-CoV-2 transmission and estimated the probability of outbreak extinction after the introduction of a single infected individual. We found that 64.1% of the model simulations exhibit stochastic extinction (defined as a realization that has no latently infected, asymptomatic, and symptomatic infectious individuals in 30 days after the introduction), with an average time to extinction of 9.6 days. Moreover, of 77.84% of the extinct outbreaks, the index case did not generate any secondary infection. In this work, following Lloyd-Smith et al.^26^, a superspreading event (SSE) is defined as any outbreak event that involves infected individuals who, on average, infect others more than the 99th percentile of the Poisson (*R*_0_) distribution. Based on this criterion, we found that 11.1% of the model realizations involve SSEs. Also, as can be seen in Fig. 4, an outbreak that involves SSEs usually spreads faster than the others.

### Evaluating the impact of interventions and heterogeneity in infectiousness on SARS-CoV-2 transmission dynamics

**Figure 5:**
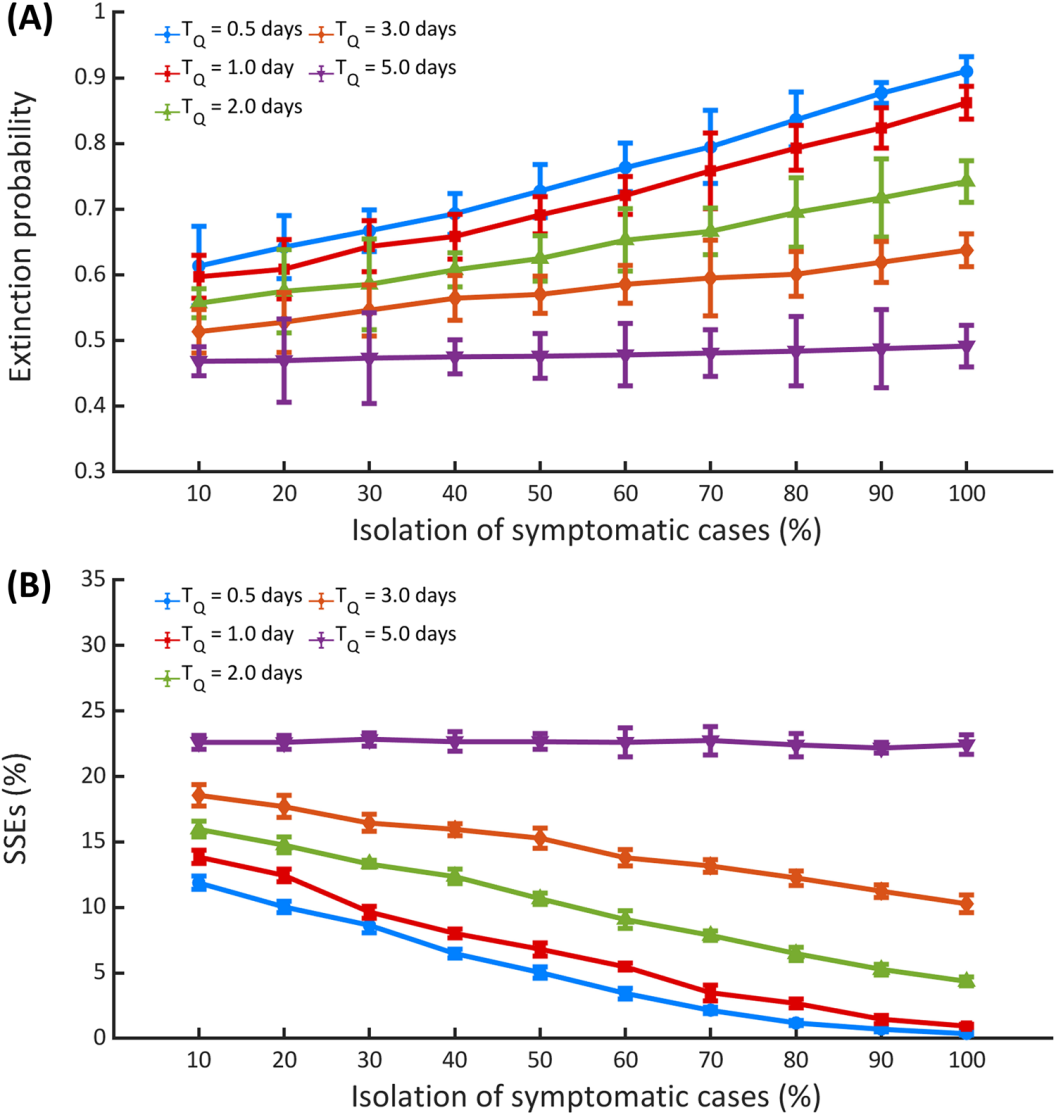
Impact of interventions. (**A**) The impact of symptomatic case isolation on the outbreak extinction (**A**) and the likelihood of SSEs (**B**). Error bars show the 95% CIs.

Next, we employed the model to evaluate the impact of interventions and individual variation in infectiousness on the SARS-CoV-2 transmission dynamics and outbreak extinction. Specifically, we explored the intervention scenarios where the mean time delay from onset of infectiousness to isolation *(*$T_{Q}$*)* is 0.5, 1.0, 2.0, 3.0, and 5.0 days together with the symptomatic case isolation ($q_{S}$) ranging from 10 to 100*%*. Our modeling results indicated that the outbreak would be more likely to become extinct if more symptomatic cases are isolated or case isolation is performed sooner (Fig. 5A). For example, more than 80% of the outbreaks will become extinct if all symptomatic cases are isolated within the first day of their infectiousness. Moreover, the isolation of symptomatic cases can also affect the likelihood of SSEs. Fast and efficient case isolation can almost eliminate the likelihood of SSEs (Fig. 5B). We also explored the intervention scenarios where the asymptomatic case isolation (*q*_*A*_) is different from the default value of 60%. In this case, we also found similar results in which the outbreak is more likely to become extinct when more asymptomatic cases are isolated (Fig. S1 in the supplementary information).

**Figure 6:**
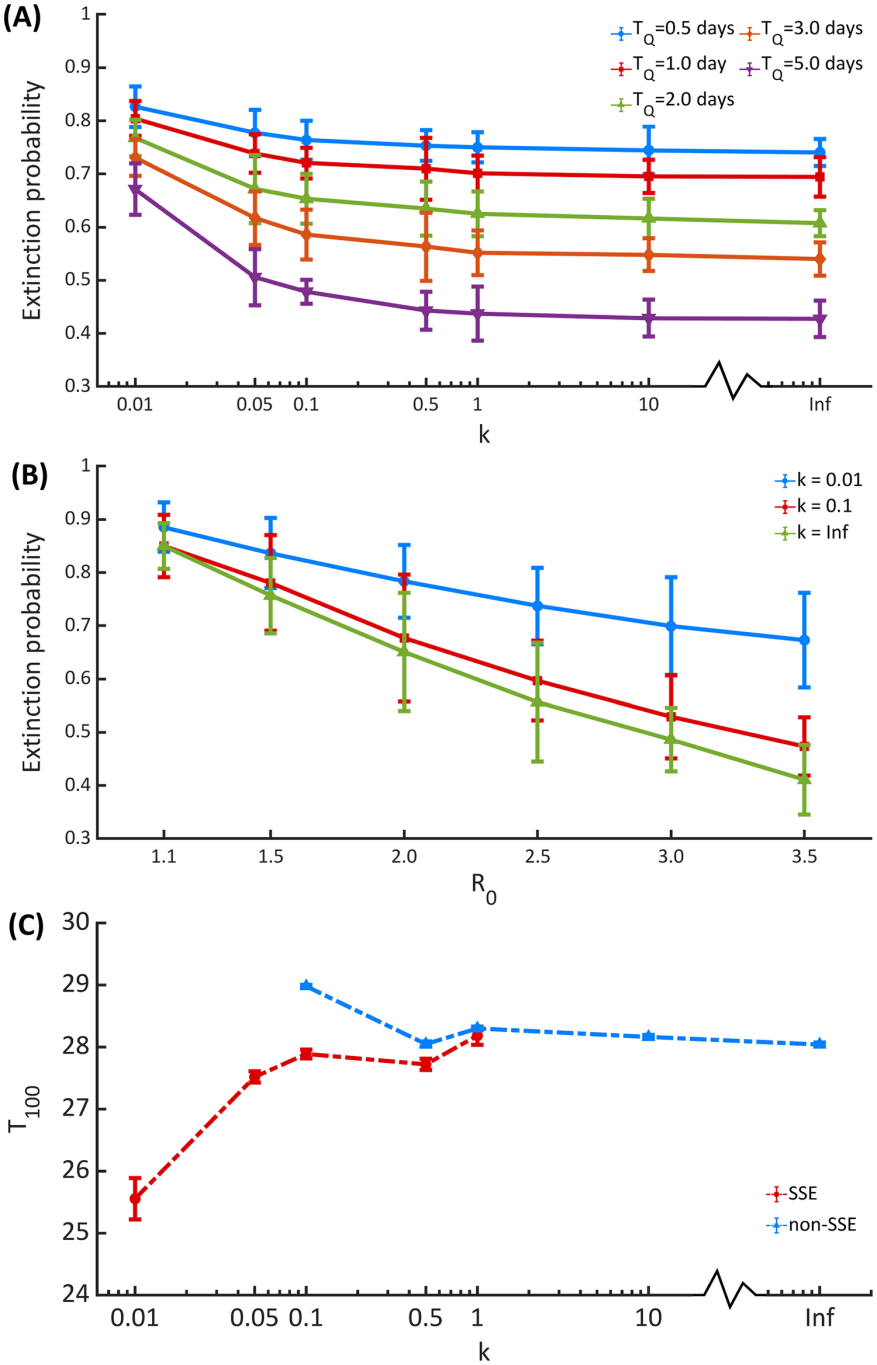
Impact of individual heterogeneity in infectiousness. The extinction probability of an outbreak with a different value of dispersion parameter $k$ (**A**) and basic reproduction number $R_{0}$ (**B**). The first date at which the cumulative number of infected individuals exceeds 100 (*T*_100_) (**C**) The results are obtained from 100,000 simulations for each parameter set. Error bars show the 95% CIs.

Figure 6A depicts the role of individual variation in infectiousness on the outbreak extinction. Here, the dispersion parameter $k$ represents the individual heterogeneity in disease transmission. A very high value of *k* indicates that each primary case generates roundly the same number of secondary cases. In contrast, a low value of *k* indicates that only a small fraction of infected individuals disproportionately infects a large number of individuals^26^. We found that individual variation on disease transmission favors the outbreak extinction, which is consistent with the previous studies^20,26–28^. Moreover, *R*_0_ can also affect the likelihood of outbreak extinction (Fig. 6B). An outbreak with a lower value of *R*_0_ is more likely to become extinct. However, lowering *R*_0_ could also reduce the impact of individual heterogeneity on SARS-CoV-2 transmission. Finally, to investigate the impact of transmission heterogeneity on the speed of disease transmission, we calculated the first date at which the cumulative number of infected individuals exceeds 100 (T_100_) (Fig. 6C). We found that an outbreak with a higher level of transmission heterogeneity spreads slightly faster. Specifically, *T*_100_ decreases from about 28 days for the homogeneous transmission ($k \to \infty$) to approximately 26 days for the transmission with a very high degree of individual heterogeneity (*k* = 0.01). Also, we found no SSE-related outbreak when $k \ge 10$. In addition, for non-SSE outbreaks with $k \le 0.05$, the cumulative number of infected individuals could not reach 100 within the simulation time of 30 days.

## Discussion

In this work, we investigated the first wave of the COVID-19 outbreak in Thailand together with the timeline of the control measures employed by the Thai government. We also estimated the time-varying reproduction number during the first epidemic wave and used stochastic simulations to reconstruct the full transmission dynamics of COVID-19 in Thailand. Recent studies have revealed important transmission features of COVID-19, including the infectiousness of asymptomatic^29,30^, individual heterogeneity in infectiousness, and SSEs^14,16,19,31^. To accurately capture the COVID-19 transmission dynamics in Thailand, these features were therefore incorporated in our stochastic event-based model. Specifically, our model assumes that both symptomatic and asymptomatic infectious individuals can transmit the disease but with different degrees of infectiousness. In addition, the expected number of secondary cases ($\nu$) caused by a primary infectious individual is assumed to follow a gamma distribution with a mean reproduction number *R* and a dispersion parameter *k*^26^.

After declaring the state of emergency and implementing the nationwide interventions, the significant decline in both the number of confirmed cases and the positive detection rate in Thailand was observed^32,33^. To evaluate the effectiveness of the control measures implemented by the Thai government during the first epidemic wave, the time-varying reproduction number, $R_{t}$, was estimated (Fig. 2). We found that after implementing the nationwide interventions, the *R*_*t*_ in Thailand was decreased from the peak value of 5.67 to a value below one in less than one month. This indicated that the control measures employed by the Thai government during the first COVID-19 epidemic wave were effective. Singapore is another country in Southeast Asia that was successful in controlling the first wave of COVID-19^34^. It was found that the mean time delay from onset of infectiousness to isolation in Singapore was about 2.6 days^35^, which is close to our estimate of 2.26 days in Thailand.

In general, mathematical modeling and computer simulation can be used either as predictive tools or as a means of understanding infectious transmission dynamics^36^. Here, the model was employed to understand the COVID-19 transmission dynamics of the first epidemic wave in Thailand. To this end, we incorporated the effects of the implemented interventions during the first COVID-19 epidemic wave by using the estimated $R_{t}$ to estimate the time-varying individual reproduction number ($\nu_{t}$) in our model^26^. We found that our model could accurately capture the transmission dynamics of the first COVID-19 epidemic wave in Thailand (Fig. 3). The model predicted that at the end of the first wave, the number of cumulative confirmed cases was 3,091 (95%CI 2782–3400), which is consistent with the reported data.

In fact, several modeling studies have been conducted to predict the incidence of COVID-19 and evaluate the effectiveness of control measures^24,33,37–39^. However, most previous models usually assumed a Poisson distribution of the number of secondary infections per infected individual, which could not capture the relevant features of superspreading events (SSEs)^12,26^. An SSE is usually defined as an event in which an infected individual transmits the disease to a large number of secondary individuals, while most infected individuals infect a few or none^20,21^. Recent studies have pointed out that only a small fraction of SARS-CoV-2 infected individuals disproportionately infect a large group of people while most infected individuals infect a few or none^20,21^. It was estimated that only 10–20% of the COVID-19 infected individuals were responsible for about 80% of transmission^12,16,26,40–42^. To accurately reconstruct the full dynamics of COVID-19 transmission, these features of disease transmission heterogeneity were therefore incorporated in our model. Specially, we assumed that the expected number of secondary cases caused by an infectious individual varies from person to person^26^.

We found that the dynamics of COVID-19 transmission depend on the degree of the individual heterogeneity in disease transmission, represented by the dispersion parameter *k*. Our modeling results indicated that individual variation on disease transmission favors the outbreak extinction, which is in line with previous studies^20,26–28^ (Fig. 6). This is because when individual heterogeneity in disease transmission is high, only a few infected individuals generate a large number of secondary infections, while most infected individuals give rise to a few or none secondary infections, leading to the higher likelihood of the outbreak extinction. However, although individual variation on disease transmission could increase the chance of outbreak extinction, it could also accelerate the transmission dynamics of the non-extinct outbreaks, which is in agreement with other studies^12,16,26^.

Our study, as with all modeling studies, has several limitations. The estimation of *R*_*t*_ was based on the reported number of confirmed cases. Although there might be potential biases due to delays in case reporting, if the delay from infection to confirmation does not change over time, this will not affect the conclusion of this study. In our study, we also assumed that the testing and reporting efforts are constant over time. However, if the testing effort increases and decreases during a particular time interval, this will increase and decrease the estimated values of *R*_*t*_, respectively^3^. In addition, although recent studies revealed that age-specific contact patterns also play a role in the COVID-19 transmission^18,43,44^, we did not incorporate them into our model; such data for Thailand is not yet available. Finally, our model did not consider migrant movements and human mobility; incorporating these data into the epidemic model might improve the modeling accuracy^45,46^.

## Materials and methods

### Data sources

The number of laboratory-confirmed COVID-19 cases in Thailand was retrieved from the Center for Systems Science and Engineering (CSSE) at Johns Hopkins University^47^. The information of implemented control measures and large outbreak events was collected from the Centre for COVID-19 Situation Administration (CCSA) of Thailand^8^.

### Duration of the first epidemic wave

The first wave of COVID-19 outbreak in Thailand was assumed to start from 22 January 2020, when the first locally transmitted COVID-19 case was reported, to 13 May 2020, which was the first date at which Thailand had no reported on COVID-19 local transmission for at least 200 consecutive days^8,11^.

### Estimation of the effective reproduction number

The effective reproduction number, $R_{t}$, is defined as the average number of secondary cases produced by an infected individual at time $t$^3^. A value of $R_{t}$ greater than the critical value of 1 indicates that the epidemic size is growing and the infection could be able to spread in the population at time $t$, whereas a value of $R_{t}$ less than 1 indicates that the epidemic size is shrinking at time *t*^3^. In this study, we employed a statistical method developed by Cori et al. to estimate $R_{t}$ during the first epidemic wave in Thailand^48,49^. This $R_{t}$ estimation method only requires the number of daily new cases and the distribution of the corresponding serial interval, which here was assumed to be a discretized Gamma distribution with a mean and the standard deviation of 6.5 days and 4.2 days, respectively^3,35,50^. Details of the *R*_*t*_ estimation are presented in ref^3^.

### Model structure

**Figure 7:**
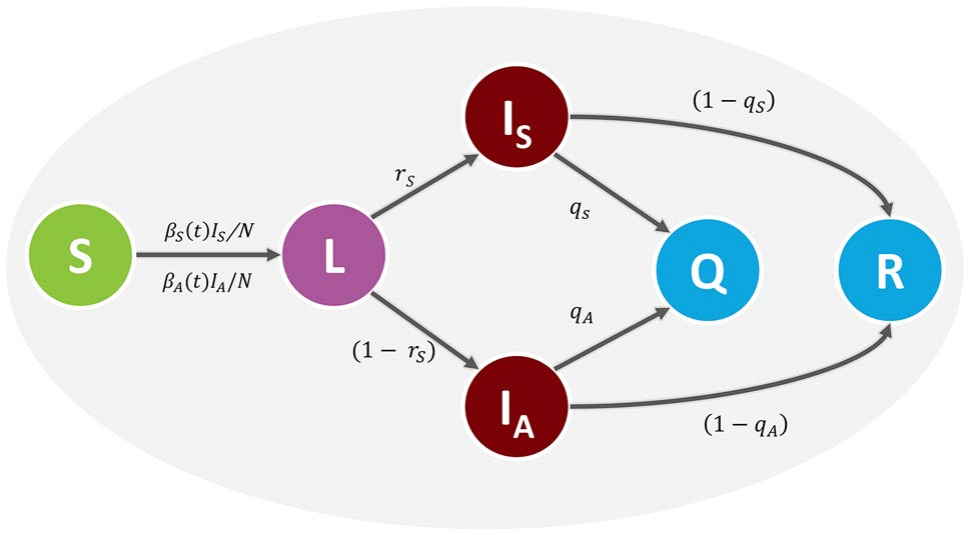
Schematic of the COVID-19 transmission model. The model comprises six epidemiological compartments: susceptible (S), latently infected (L), symptomatic infectious (I_S_), asymptomatic infectious (I_A_), quarantined (Q), and recovered (R).

A stochastic event-based modeling approach was employed in this study. The schematic of the model specification is shown in Fig. 7. The model divides the population into six separated compartments; namely, susceptible (S), latently infected (L), symptomatic infectious (I_S_), asymptomatic infectious (I_A_), quarantined (Q), and recovered (R) compartments. A susceptible individual can get an infection from either a symptomatic or an asymptomatic infectious individual at rates $\beta_{S}$ and $\beta_{A}$, respectively. After being infected, the susceptible individual progresses to the latently infected class. Individuals in this class have already acquired the SARS-CoV-2 infection but are not yet infectious and cannot transmit the disease to other susceptible individuals. Latently infected individuals become infectious at a rate that is inversely proportional to the mean latent period (*T*_*L*_). A proportion $r_{S}$ of the infected individuals becomes symptomatic infectious individuals, while the remaining $1 - r_{S}$ becomes asymptomatic infectious individuals. In addition, a proportion *q*_*S*_ of symptomatic infectious individuals and a proportion *q*_*A*_ of asymptomatic infectious individuals is isolated and quarantined at a rate that is inversely propositional to the mean time delay from onset of infectiousness to isolation *(T*_*Q*_*)* while the remaining is recovered at a rate that is inversely proportional the mean infectious period *(T*_*I*_). We assumed that all isolated individuals could not make a further transmission.

We incorporated the disease transmission heterogeneity into the model by assuming that the expected number of secondary cases caused by an infectious individual varies from person to person^26^. In our work, the individual reproduction number ($\nu$) of each infectious individual was assumed to follow a gamma distribution with mean $R_{0}$ and dispersion parameter $k$, $\nu \sim {\text{gamma}}\left( {R_{0} ,k} \right)$, where $R_{0}$ is the basic reproduction number^12,26^. The transmission rates due to symptomatic infectious individuals ($\beta_{S}$) and asymptomatic infectious individuals ($\beta_{A}$) are given by

1 $$\beta_{S} = q_{S} \gamma_{S} \nu_{Q} + \left( {1 - q_{S} } \right)\gamma_{S} \nu ,$$

2 $$\beta_{A} = q_{A} \gamma_{A} \nu_{Q} + \left( {1 - q_{A} } \right)\gamma_{A} \nu ,$$

where $\nu_{Q} = \left( {T_{Q} /T_{I} } \right)\nu$ is the expected number of secondary cases generated by a quarantined individual before isolation. Here we assumed that $\nu_{Q}$ is linearly proportional to the mean time delay from onset of infectiousness to isolation *(T*_*Q*_*).* $\gamma_{S}$ and $\gamma_{A}$ are the symptomatic and asymptomatic recovery rates that are inversely proportional to the mean infectious period.

The total number of infection events that occurred during the time interval *dt* is given by

3 $$N_{T} \left( t \right) = {\text{Poisson}}\left( {\mathop \sum \limits_{j = 1}^{{n_{I} }} \frac{{\beta_{i}^{j} Sdt}}{N}} \right),$$

where $n_{I}$ is the total number of infectious individuals at time $t$*,* and $i = S$ or $A$ representing symptomatic or asymptomatic infectious individual, respectively. The numbers of all other transition events during the time interval *dt* were calculated using the standard event-based modeling algorithm and $\tau$-leap method^51^.

The model simulations begin with a single infectious individual at time *t* = 0. For each set of parameters, we simulated 10 batches of 1,000 model realizations. Following Lloyd-Smith et al.^26^, a model realization that involves infected individuals who, on average, infect others more than the 99th percentile of the Poisson *(R*_0_*)* distribution is classified as an SSE-related outbreak. An outbreak extinction was defined as a realization that has no latently infected, asymptomatic, and symptomatic infectious individuals in 30 days after the introduction of the index case. The model simulations were implemented in MATLAB R2019b. The parameters and their default values used in the model are summarized in Table 1.

**Table 1:** Descriptions and values of all parameters used in the model.

Parameter	Definition	Value	References
$T_{L}$	Latent period	4.0 days	^29,35,52^
$r_{S}$	Proportion of infected individuals who are eventually symptomatic	0.6	^35,50,53^
*T*_*I*_	Infectious period	5.0 days	^29,35,54^
$T_{Q}$	Time delay from onset of infectiousness to isolation	Varied	
$\gamma_{S}$	Symptomatic recovery rate	1/5 day^−1^	
$\gamma_{A}$	Asymptomatic recovery rate	1/5 day^−1^	
$q_{S}$	Proportion of symptomatic infectious individuals who are isolated and quarantined	Varied	
$q_{A}$	Proportion of asymptomatic infectious individuals who are isolated and quarantined	0.6	^55^
$k$	Dispersion parameter	Varied	
$R_{0}$	Basic reproduction number	2.2	^56^
$N$	Thailand population size	$6.943 \times 10^{7}$	

### Reconstruction of the COVID-19 transmission dynamics in Thailand

To reconstruct the transmission dynamics of COVID-19 in Thailand, we used the estimated $R_{t}$ during the first epidemic wave in Thailand to estimate the time-varying individual reproduction number ($\nu_{t}$). Specifically, $\nu_{t}$ was drawn from the gamma distribution with mean $R_{t}$ and dispersion parameter $k$, $\nu_{t} \sim {\text{gamma}}\left( {R_{t} ,k} \right)$. The model structure employed in this section is the same as the one described in the Model structure section, except that the individual reproduction number here is time-dependent. In addition, based on the surveillance information, the initial number of symptomatic infectious individuals was set at five*,* while the initial numbers of latently infected, quarantined, recovered, and asymptomatic infectious individuals were set at zero.

## Supplementary Information

Supplementary Information.

## Data Availability

The authors declare that the data supporting the findings of this study are available within the paper.
