# Supplementary material for: Reconstruction of the transmission dynamics of the first COVID-19 epidemic wave in Thailand
Source: Sci Rep. 2022 Feb 7;12:2002. doi: 10.1038/s41598-022-06008-x (PMC8821624; doi:10.1038/s41598-022-06008-x)
Supplement: Supplementary file 1 — Supplementary Information. [file 41598_2022_6008_MOESM1_ESM.pdf]

Supplementary Information:

Reconstruction of the transmission dynamics of the first COVID-19 epidemic wave in Thailand

Chaiwat Wilasang[1], Natcha C. Jitsuk[1], Chayanin Sararat[1], and Charin Modchang[1,2,3*]

[1] Biophysics Group, Department of Physics, Faculty of Science, Mahidol University, Bangkok 10400, Thailand

[2]Centre of Excellence in Mathematics, CHE, Bangkok 10400, Thailand

[3]Thailand Center of Excellence in Physics, CHE, 328 Si Ayutthaya Road, Bangkok 10400, Thailand

* To whom correspondence should be addressed. Email : charin.mod@mahidol.edu

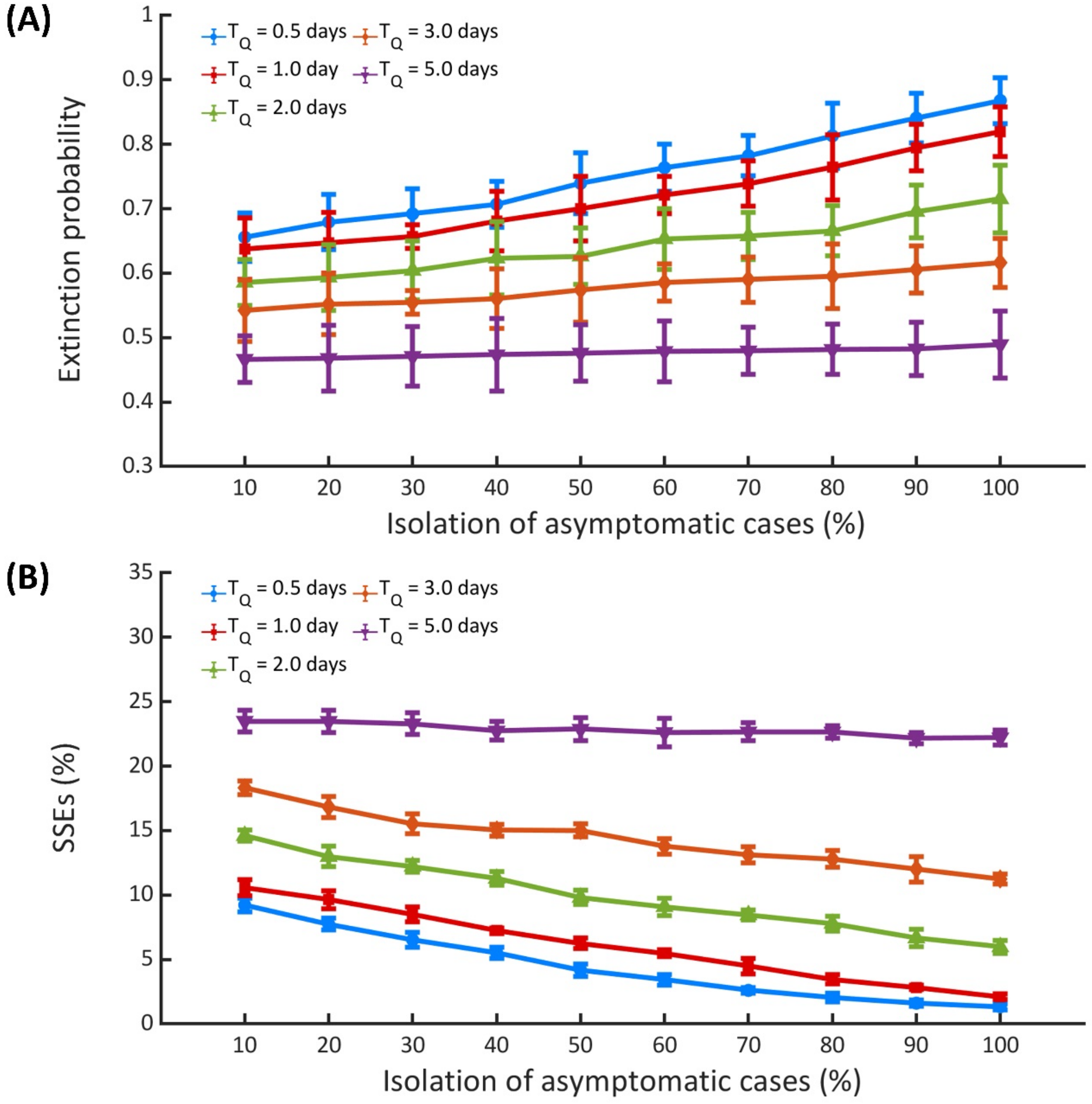

Figure S1. Impact of interventions. (A) The effect of asymptomatic case isolation on the outbreak extinction **(A)** and the likelihood of SSEs **(B)**. The proportion of isolated symptomatic infectious individuals (q_S) was fixed at 60%. Error bars show the 95% CIs.
